# Mr. Cuming, on Dysentery

**Published:** 1804-12-01

**Authors:** Ralph Cuming

**Affiliations:** Romsey


### Medical and Physical Journal.

VOL. XII.] DECEMBER 1, 1804. [NO. LXX.

*Printed for R. PHILLIPS, by W. Thorne, Red Lion Court, Fleet Street, London.*

### *To the Editors of the Medical and Physical Journal.*

GENTLEMEN,

IT often happens that those medicines made use of in the common routine of practice, which, generally speaking, answer our purpose in the established methodus curandi, in certain diseases, are frequently, from unforeseen and fortuitous circumstances, rendered totally inert. Having lately experienced an instance of this kind, I am induced, with your permission, to give it that publicity which the Medical Journal will afford it, from a thorough conviction that a faithful narration of similar occurrences, will prove conducive to the good of the community, by adding to our present stock of knowledge in therapeutics, and by putting us in possession of a dernier resource, when every other method has proved abortive; for such knowledge will prove a real treasure, capable of wearing down the rough edge of disappointment, and soothing that anxiety which every professional man must feel for the welfare of his patient, as well as his own reputation; which are desiderata of no small magnitude to a man endued with *common* sensibility. Our motto ought to be, Ne laissez rien à tenter.

Having at present the care of the paupers of this place, I was desired the other day, to visit a young man who had been subject to a violent purging for some time previous to my attendance. I found him affected with many of the symptoms of typhus gravior; he was comatose, frequently muttered, and occasionally catched at the bedclothes; his tongue was covered with a brown fur, his skin hot, pulse slow and feeble. The period for cleansing and preparing the primæ viæ for the exhibition of necessary remedies had long elapsed, and the only plan of cure which I thought could be adopted with any prospect of success, success, was at once to endeavour to check the purging, and restore perspiration. To effect these purposes I administered the pulv. ipec. comp. beginning with ten grains three times a day, and increasing the dose till he took two scruples in twenty-four hours; he was put into the hot bath, and had afterwards flannel applied to his body; his food was farinaceous and gelatinous, but his stomach was so irritable, it could retain little, and that quickly passed through him; his egesta were often tinged with blood. I considered his disease to be a well marked dysentery, as it was attended with tenesmus and shooting pains in his bowels, particularly about the regio umbilici. Finding those remedies fail, I had recourse to a variety of astringents, such as catechu & cret. pp. combined with aromatics, and laudanum to the amount of gt. 100 in the course of the day and night; but these were also useless, and did not appear to alleviate a single symptom. I was hitherto completely baffled, and now determined to try the effects of solid opium; and, strange to tell, he took seven grains in twenty-four hours, without restraining the purging. Though I have been in the habit of treating dysentery in tropical climes, in the whole course of my practice I never knew opium have so little effect: it has frequently cured this complaint, I may almost say, per se; and with the assistance of a saline purgative or two, it seldom failed; but my patient in this case was so much reduced, that this practice might have been considered as an act of unpardonable temerity. Nor durst I attempt to give a combination of calomel with opium; I therefore had recourse to mercurial inunction, and with the happiest effects it crowned the earnest expectations I formed from it. About two ounces of the ungt. fort. were rubbed in, before its operation on the system became visible. As soon as his mouth was affected, his skin grew moist; and on visiting him one morning, I was astonished to find him in a perspiration so profuse, that the hair of his head resembled the grass in a summer's morning, bespangled with dew. From hence we may conclude, that the hair which people lose after fevers, is occasioned from lack of moisture.

Every untoward symptom now began to vanish; his mind, which had been so long oppressed, again exerted its rational powers; his stomach, instead of loathing food, began to relish it; his purging soon left him; in fine, the stimulating and deobstruent qualities of the mercury completely restored the healthful equilibrium, which appeared to have been destroyed from want of cuticular secretion; and and gentle tonics, assisted by nourishing diet, soon restored him to his wonted health.

I am perfectly convinced, that a more general adoption of those methods of cure which have of late obtained in the *public service*, would prove highly beneficial to private practitioners, and the public at large; but the ever to be deprecated idea, that innovations are *always* dangerous, serves to shut the door of reason, and prevent the cheering rays of science and improvement from illuminating the benighted mind. The good effects of mercurial inunction in typhus and malignant fevers, have been displayed in numberless instances, and well merit the most serious attention of such medical gentlemen as are strangers to this mode of practice: and I should not be surprised to hear, that this treatment has been the most successful, in the cure of that terrible scourge which is at present committing such ravages at Gibraltar.

I lately, through the medium of your Journal, when treating on Sphacelus, made mention of the powerful effects of nitre, in correcting putrescency, and arresting the progress of mortification; two cases were then adduced, where its efficacy was proved in the most unequivocal manner. I have at this moment a patient under my care, who had a mortificatiou on the external part of the fore-arm and hand, occupying a space of 9 inches in length. The integuments, fascia, and part of the muscles were completely destroyed, as well as the extensor digiti medii tendon; and though the mortification was so extensive, the repeated application of powdered nitre prevented the mass of humours from being corrupted by the absorption of putrid virus; it appeared very evident at every dressing, that the nitre had perfect command over this dreadful disease, by entirely subduing that cadaverous fœtor which always exhales from mortified parts. When I found the smell no longer offensive, I applied a cataplasm of roasted onions to the part, by way of removing the slough, and assisted its operation by means of fomentations, composed of bitter and antiseptic herbs, such as wormwood, &c. The line of separation soon appeared, and the sphacelated parts were easily removed, (the tendon excepted, which was a considerable time in sloughing away) when the onions were discontinued, and boiled carrots were applied; the wound soon began to assume a favourable aspect, being filled up with healthy florid granulations, and is now nearly cicatrized.

 It

It is worthy of remark, that the patient's stomach was very little affected; he was enabled to take in a sufficient quantity of nutriment, which, in all probability, would not have been the case, if absorption had taken place, or his stomach had been loaded with *huge doses of bark*; he took the decoct. cinchonæ, and was allowed strong beef soup, with a liberal quantity of port wine and mild ale. Dr. Willich, in his Treatise on Diet and Regimen, has remarked, that pork predisposes to mortification; and I believe there is much truth in the observation. My patient had subsisted chiefly on pig-meat; and it appears from the history of his case, that his misfortune arose, in the first instance, from a small pimple on his hand, which became so troublesome, that he was induced to apply to a celebrated empiric, whose skill was so perfectly baffled in this case, that he was constrained to acknowledge, from the black, blistered, swelled, and frightful appearance which the patient's hand and arm exhibited, that the case required more of the acumen chirurgicum, than he could boast of, and recommended his poor suffering and deluded patient to apply for further advice, as mortification had already taken place. Nor was it surprising, considering the treatment which had been adopted; white lily root and a variety of acrid substances were applied to assuage a most active inflammation! As I have so frequently experienced the good effects of the remedy which I so strenuously recommend, I request as a *particular favor*, that some of my medical brethren will, when opportunity offers, publish the result of cases which may occur in their practice, should they be disposed to place that confidence in it which its efficacy so justly entitles it to. In a crowded hospital, after a great naval action, I have known some of the stumps become sphacelous; but these unhappy men soon fell victims to death: I have since often regretted, that the virtues of this invaluable medicine were then unknown to me.

It has lately been ascertained, that the nitrous acid gas possesses the power of destroying contagion, on which account Government supplies the Navy with the materials and utensils necessary for the purpose of fumigation. It has often been proved, that malignant ulcers, exposed to the action of the fumes, have been converted into a mild state, and easily cured: yet, having a knowledge of these circumstances, I must confess that I was not induced to use this remedy from any analogical inference striking my mind, but from the simple idea, after a fruitless struggle gle to subdue the fœtor issuing from a sphacelous foot, that as nitre was found to preserve animal substances from putrefaction, it might also arrest its progress when commenced; and such has been invariably the result in the cases which have come under my care.

It may not be amiss to inquire into the modus operandi of this medicine, which I shall only attempt in a cursory manner, and leave the rest to those who are more fond of speculative and chemical inquiries than I am.

Modern chemists have discovered, that nitre is a composition of oxygen and azote; and whatever may be the component parts of animal substances in a state of putrescency, it is certain they contain some part very greedy of oxygen, which is readily imparted by the penetrative and soluble nature of this salt, that seems to neutralize and render the offensive mass perfectly innocuous. Charcoal, which is said to contain a quantity of oxygen, has frequently been applied to mortified parts; but it is probable that it cannot give it out with the same facility as nitre does; on which account it is less efficacious. Though this line of the poet be not applicable, "Omne tulit punctum, qui miscuit utile *dulce*," I hope the request I have made may be attended to, and that the time I have employed on this paper will not be considered as mispent.

I am, &c.

